# The National Organ Donation and Transplantation Program in Greece: Gap Analysis and Recommendations for Change

**DOI:** 10.3389/ti.2023.11013

**Published:** 2023-05-25

**Authors:** Charlotte Johnston-Webber, Apostolos Prionas, George Wharton, Simon Streit, Jasmine Mah, Ioannis Boletis, Elias Mossialos, Vassilios Papalois

**Affiliations:** ^1^ Department of Health Policy, London School of Economics and Political Science, London, United Kingdom; ^2^ Department of Surgery, Imperial College, London, United Kingdom; ^3^ Department of General Surgery, Whipps Cross Hospital, Barts Health NHS Trust, London, United Kingdom; ^4^ Department of Medicine, Dalhousie University, Halifax, NS, Canada; ^5^ Department of Nephrology and Kidney Transplantation, General Hospital of Athens Laiko, National and Kapodistrian University of Athens, Athens, Greece; ^6^ Institute of Global Health Innovation, Imperial College, London, United Kingdom; ^7^ Renal and Transplant Unit, Hammersmith Hospital, Imperial College Healthcare NHS Trust, London, United Kingdom

**Keywords:** organ donation, organ transplantation, transplantation policy, transplant system, Greece

## Abstract

Greece has fallen far behind many comparable European countries in the field of organ donation and transplantation and has made little progress over the past decade. Despite efforts to improve its organ donation and transplantation program, systemic problems persist. In 2019, the Onassis Foundation commissioned a report to be prepared by the London School of Economics and Political Science that focused on the state of the Greek organ donation and transplantation program and proposed recommendations for its improvement. In this paper, we present our analysis of the Greek organ donation and transplantation program together with an overview of our specific recommendations. The analysis of the Greek program was undertaken in an iterative manner using a conceptual framework of best practices developed specifically for this project. Our findings were further developed *via* an iterative process with information provided by key Greek stakeholders and comparisons with case studies that featured successful donation and transplantation programs in Croatia, Italy, Portugal, Spain, and the United Kingdom. Because of their overall complexity, we used a systems-level approach to generate comprehensive and far-reaching recommendations to address the difficulties currently experienced by the Greek organ donation and transplantation program.

## Graphical Abstract

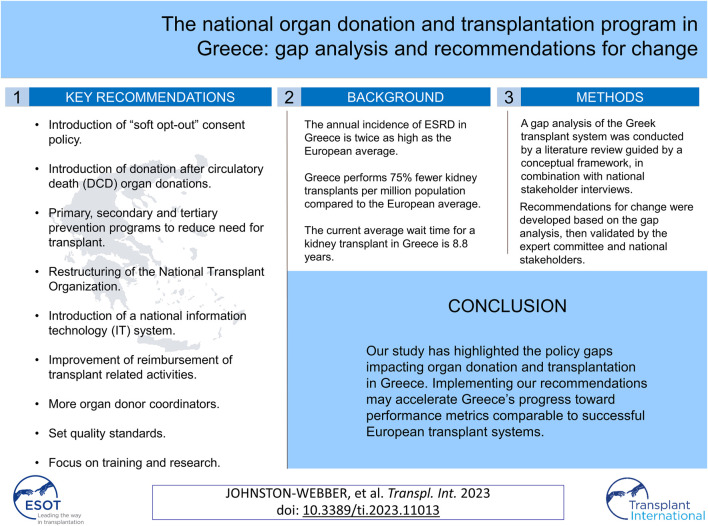

## Introduction

The fields of transplant medicine and surgery have experienced extraordinary advances over the past 50 years. Despite this progress, the process of establishing an efficient and effective organ donation and transplantation program remains a challenge for many countries. Given the extensive scope of the many processes involved, for example, the design of both consent policies and clinical protocols, efforts to develop a successful program that meets the needs of a specific population are by their nature complex and require ongoing commitment, investment, evaluation, and improvement. Given the magnitude of the returns, both in terms of economics and quality of life, the case for investing in a comprehensive organ donation and transplantation service is certainly indisputable.

Chronic kidney disease accounts for most cases of organ failure that ultimately lead to transplantation. Greece has among the highest incidences of end-stage renal disease (ESRD) among high-income countries. This outcome is driven by high rates of smoking, obesity, and poor cardiovascular health (1, 2). As a result, there are on average twice as many new dialysis patients per million population (pmp) each year in Greece compared to rates reported in other European countries. For example, in 2017, there were 1,319 patients pmp in Greece who were diagnosed with ESRD. This is far above the European average of 854 patients pmp. The annual incidence of ESRD in Greece is typically twice as high as the European average. In 2017, 252 patients pmp in Greece began renal replacement therapy compared to the European average of 127 patients pmp (3). Other demographic characteristics, notably Greece’s aging population together with the overall lack of control over modifiable risk factors will likely increase the incidence of chronic diseases, and hence the need for organ transplants (1, 2, 4).

Although national healthcare expenditure *per capita* in Greece is somewhat below the European average (Table 1), it is similar to the *per capita* healthcare spending of several less well-endowed European countries that have developed successful transplantation systems, including Croatia and Portugal (5). Greece was severely affected by the 2008 economic crisis; austerity measures that were imposed at that time resulted in significant cuts to healthcare spending. From 2012 to 2017, the proportion of the gross domestic product (GDP) spent on healthcare in Greece decreased by 9.4% (5). However, other similarly affected countries, including Spain, Portugal, and Croatia have since developed and maintained successful organ donation and transplantation programs (6–10).

**TABLE 1:** Health system financing and population health in Greece: key statistics.

Health system	References
• Highly centralized mixed health system model with a single health insurer	(2)
• Health spending *per capita*, EUR 1603; EU average, EUR 3523	(2)
• Health spending as a percentage of the GDP, 7.8%; EU average, 9.9%	(2)
• Public spending as a percentage of the total health expenditure, 60%; EU average, 80%	(2)
• Out-of-pocket payments as a percentage of the total health expenditure, 35%; EU average, 15.4%	(2)
• Percentage of the population reporting an unmet need for medical care, 8.1%; EU average, 1.7%	(2)
**Health status**
• Percentage of the population over 65 years of age, 22.3%; EU average, 20.6%	(2)
• Life expectancy, 81.2 years; EU average, 80.6 years	(2)
• Percentage of the population that smokes daily, 24.9%; OECD average, 16.5%	(11)
• Liters of alcohol consumed *per capita* per year, 6.3L; OECD average, 8.7L	(11)
• Percentage of the population that is overweight or obese (BMI >25), 57.2%; OECD average, 56.4%	(11)
• Individuals maintained on renal replacement therapy; incidence, 269 pmp	(12)
• Individuals maintained on renal replacement therapy; prevalence, 1413 pmp	(12)

EUR, Euro; EU, European Union; OECD, Organisation for Economic Co-operation and Development; BMI, body mass index; GDP, Gross Domestic Product.

There is thus an undeniable need for a more comprehensive and effective organ donation and transplantation program in Greece. However, despite a disproportionally high level of clinical need, Greece lags far behind European countries with respect to rates of organ donation and transplantation. Between 2008 and 2019, Greece reported between 4.1 and 8.9 donors pmp each year (13-24). During the same period, the number of deceased donations in Europe as a whole increased steadily from 10.7 pmp in 2008 to 17.13 pmp in 2019 (25); many countries out-performed these averages. Greece performs 75% fewer kidney transplants pmp compared to the European average and has the lowest rates of transplantation among members of the Organization for Economic Co-operation and Development (OECD). Furthermore, transplantation rates in Greece have stagnated in recent years (25). According to the Greek National Transplant Organization (NTO) known as the Hellenic Transplant Organization (EOM), the current average wait time for a kidney transplant in Greece is 8.8 years. The average survival of patients that have started on dialysis is 3 years and that one-quarter of these patients will die within 1 year (26).

This paper aims to review the policies on solid organ donation and transplantation in Greece and to evaluate the current performance of its national program. We will also present a brief overview of our recommendations for improvement and reform.

## Materials and Methods

**FIGURE 1:**
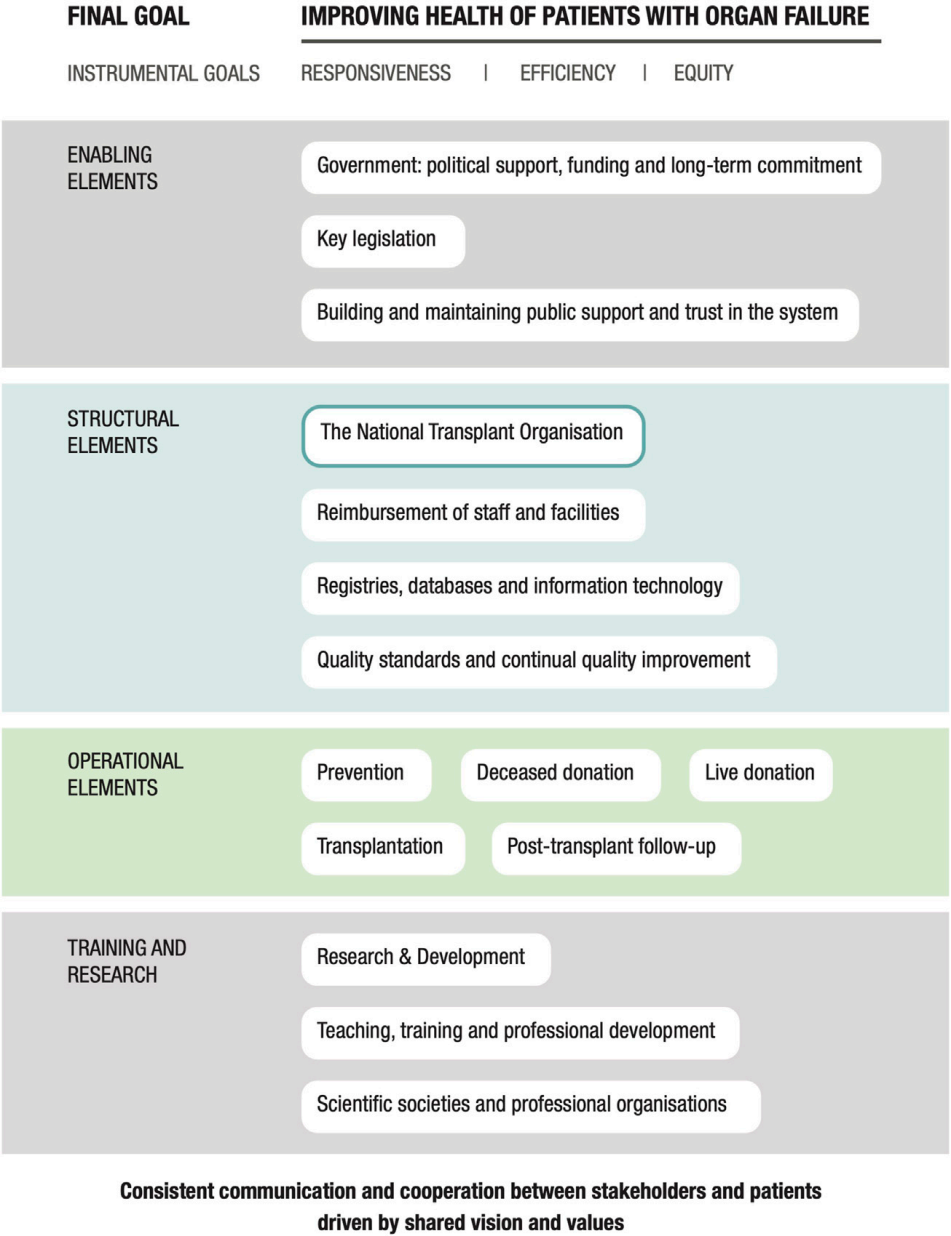
A conceptual framework for a successful national organ donation and transplantation system.

Two main steps were involved in the development of this study. First, we performed a gap analysis of the Greek organ donation and transplantation program that was guided by a conceptual framework of best practices (Figure 1) and further informed by case studies that focused on five successful national organ donation and transplantation programs in other European countries (6–10). We also conducted an extensive series of interviews with Greek stakeholders and international experts from a wide range of sectors that are relevant to organ donation and transplantation. Recommendations for reform of the Greek program were generated based on the results of the gap analysis with additional input from the panel of Greek and international experts. The full list of expert participants is included in Supplementary Appendix SA2.

### Gap Analysis

We began by conducting a brief narrative review of the relevant peer- and non-peer-reviewed literature. The findings were supplemented with information collected from focused interviews with global, national, and local policy experts (27, 28). The literature search and interview questions were based on a conceptual framework that was developed previously by the research team that highlights the essential building blocks of a successful national solid organ donation and transplantation program (Figure 1) (29). The analysis focused on structures, processes and distinctive features of the system corresponding to domains of the framework, rather than performance in relation to health outcomes or health system goals.

Peer-reviewed articles were retrieved from searches of EconLit, MEDLINE (PubMed), Embase (Ovid), Scopus, and Web of Science using several relevant search terms. We restricted our search to papers published between 1 January 1968, and 26 February 2021, that were written in either English or Greek, and included journal research articles, comments, editorials, and reviews. Following the initial compilation of the search results and removal of duplicate entries, we then excluded papers that focused on topics other than solid organ donation and transplantation. We ultimately identified 15 unique peer-reviewed papers that focused specifically on solid organ donation and transplantation in Greece (See Supplementary Appendix SA1). A selection of relevant non-peer-reviewed (i.e., grey) literature was identified *via* a Google search and from citations in key published papers. In total, our study incorporated results from 29 non-peer reviewed texts that included policy documents, working papers, conference presentations, and consulting reports.

A series of hour-long telephone interviews were conducted with a panel of 25 expert stakeholders in the field of solid organ donation and transplantation. These individuals were asked to validate and provide information that was complementary to the conclusions drawn from the literature review. A combination of convenience and judgment sampling was used to select interviewees who were accessible and might have professional insight into the policy, clinical, ethical, political, media, and regulatory environment of solid organ donation and transplantation (27). The full list of interviewees is included in Supplementary Appendix SA2. We also solicited input from the Greek Ministry of Health, the NTO, and several patient associations.

A semi-structured interview protocol was employed that covered all aspects of solid organ donation and transplantation in Greece, including regulations as well as current and prospective future policies. Time was also provided for unstructured dialog focused on relevant topics.

### Recommendations

This case study differs from the others in this series as it includes an additional focus on policy recommendations that might be used to improve the Greek national organ donation and transplantation program. Consensus on the recommendations for the reform and development of the Greek program was reached *via* a structured iterative process. As part of the first step, the research team used the results of the gap analysis to develop a set of preliminary recommendations to provide Greece with a framework to address the gaps and achieve high performance in each area. The project co-chairs then reviewed these recommendations and made several suggestions and refinements. The views of the stakeholder panel were then sought, which generated additional feedback and suggestions. The objective was to elicit feedback from as many disciplines as possible; thus, broad categories of stakeholders consulted included representatives of the NTO, national transplant centers, intensive therapy units, scientific societies, professional bodies, patient associations, the national bioethics committee, national representatives to the World Health Organization (WHO), political authorities, and the press. The research team integrated this feedback with the initial set of recommendations and undertook an additional cycle of review and refinement. The resulting set of recommendations was shared with the international and Greek panels of experts who submitted written feedback and participated in online round-table meetings to provide further comments. These comments were incorporated into a final set of recommendations.

## Results

Greece has a long history of solid organ transplantation. The information shown below in Table 2 summarizes some of the key developments and trends in organ donation and transplantation in Greece since 1968.

**TABLE 2:** Key developments and trends in organ donation and transplantation in Greece.

1968	The first successful deceased donor renal transplant was performed
1972	The first renal transplant program was developed (30–32)
1978–1987	Legislation was gradually introduced that provided a framework and facilitated transplantation activities, including legislation focused on the definition and diagnosis of brain death and the processes to be used for organ donation, retrieval, and transplantation (30–32)
1990–1993	Programs for liver, heart, and pancreas transplants were established (30–32)
1990s–early 2000s	Extensive legislation was introduced that defined all the dimensions of the transplantation sector
2001	The Greek National Transplant Organization (NTO) was founded
2005	An Organ Donation Coordinator (ODC) program was launched as part of the attempt to adopt the “Spanish Model”
2008	Lung transplant programs were initiated (30–32)
2008	Transplantation activity in Greece reached its peak with 28 solid organ transplants performed per million population (pmp). This approached the contemporary European average of ∼35 solid organ transplants pmp (22)
2011	Lung and pancreas transplant programs were discontinued (30-32)
2011	A sharp decline in organ donation and transplantation rates was observed. The government instituted major legislative changes including the introduction of an opt-out consent system(30–32)
2013	The opt-out consent system was implemented
2014	A unified and centrally-governed organ allocation system and a national renal transplant registry were introduced (30–32)
2015	A historic low of 11 solid organ transplants pmp was reported. This is less than a quarter of the European average of 46 solid organ transplants pmp reported at this time (25)
2018	An opt-in consent policy was re-introduced (33–37)

### Gap Analysis

The following sections provide an overview of the main areas in which the organ donation and transplantation program in Greece currently falls short of internationally-recognized standards and highlight areas with the greatest potential for improvement. These results are presented in Table 3 according to the domains and key elements of the conceptual framework.

**TABLE 3:** Key areas highlighted by the gap analysis in which significant shortfalls in the Greek organ donation and transplantation program were identified.

Framework domain	Key features	Details
Enabling Elements: Government: Political Support, Funding, and Long-Term Commitment	Funding	Lack of financial stability and long-term sustainability
	Government commitment	Lack of strategic planning and continuity
Enabling Elements: Government: Key Legislation	Diagnosis of brain death	Transplant legislation has defined brain death. This gives the false impression of conflicts of interest. There are no clear provisions regarding the performance of diagnostic tests or the role of ancillary testing
	Modes of donation	Pediatric live donation (LD) is prohibited. There are no provisions for adult or pediatric controlled or uncontrolled donation after circulatory death (cDCD/uDCD), withdrawal of life-saving treatment (WLST), “no-touch” time, or directed or non-directed altruistic donation
	Consent policy	An opt-in consent system is in effect
Enabling Elements: Building and Maintaining Public Support and Trust in the System	Creating public trust	There are high levels of public distrust in the system
	Periodic surveys and educational campaigns for the general public	Public awareness of organ donation is limited
	Communication strategies	There are no targeted national communication strategies
Structural Elements: National Transplant Organization	Leadership capacity	The National Transplant Organization (NTO) has no authority to enact reforms in the system
	Organizational structure	The current organizational structure does not correspond with the responsibilities defined by national legislation
	Organizational resources	The program is short-staffed and under-resourced
	National and international responsibilities	The responsibilities defined by legislation follow international best practices. However, the organization’s national responsibilities are not met and few international collaborations have been established
Structural Elements: Infrastructure	Workforce	The numbers of transplant surgeons, physicians, and anesthetists are below the European average
	Facilities	There are five transplant centers in Greece. One new transplant center is currently under construction. The goal is to accommodate additional pediatric and adult transplant services. The capacity of the operative theaters and access to imaging, endoscopy, pathology, and histocompatibility services are below the European standard at all five of the existing transplant centers
Structural Elements: Reimbursement of Staff and Facilities	Mechanisms of reimbursement	The KEN-DRG diagnosis-related group reimbursement system covers transplantation activities but not activities related to donation (e.g., maintenance of donors in the intensive care unit, organ retrieval, among other activities). There is no provision for reimbursement of organ donor coordinators for work specifically related to donation
	Incentives	Donation activities represent a considerable financial burden to participating hospitals
Structural Elements: Registries, Databases and Information Technology (IT)	Registries and waiting lists	There are no living donation (LD) registries
	IT and data protection	Critical donor data are not easily accessed by the different parties involved due to the absence of a functional IT system
Structural Elements: Quality Standards and Continual Quality Improvement	Maintaining quality standards	While quality standards for authorization and licensing of transplant units are clearly defined in national legislation, there is no evidence of regular inspection of transplant facilities, equipment, or personnel. There are no quality standards provided to guide donation, pre-transplant, or post-transplant care. A few quality indicators are monitored annually by the NTO.
	Driving quality improvement	Although performance data comparing transplant centers are published every 3 years, there are no nationally agreed-upon procedures designed to facilitate improvement. No evidence of quality improvement interventions was found
Operational Elements: Prevention	Primary prevention	There are no public health programs designed to prevent heart, lung, liver, and kidney disease
	Secondary prevention	There are no screening programs targeting populations at high risk of developing heart, lung, liver, and/or kidney disease
Operational Elements: Deceased Donation	Donation coordination	Although the qualifications, training, duties, and responsibilities of organ donation coordinators (ODCs) are defined by national legislation, there is no legislative provision for protected time to perform duties or any form of financial reimbursement. Most of the ODC posts in Greece remain unfilled
	Donor evaluation and management	There are no nationally agreed-upon guidelines for the evaluation and management of deceased donors
	Organ retrieval, preservation, and transport	There are no nationally agreed-upon protocols for organ retrieval, preservation, or transport
Operational Elements: Live Donation (LD)	Promoting LD	Although there are legislative provisions that address reimbursement of living donors for costs incurred, there are currently no policies that promote living donation
	Assessment of living donors	There are no nationally agreed-upon guidelines for the evaluation and management of living donors
Operational Elements: Transplantation	Referral and assessment for transplant	There are no nationally agreed-upon criteria or standardized processes to guide patient referrals and assessments for suitability for transplant. There are also no nationally agreed-upon criteria for listing decisions
	Transplant coordination	Only seven transplant recipient coordinators, all of whom are all based at the NTO headquarters, are responsible for the coordination of all transplant procedures throughout the entire country. Because of staff shortages, this service is not available on all days or at all times
	Surgery and perioperative care	The lack of physical and human resources hinders timely access to operating theaters. There are no standardized national peri-operative care protocols
	Access to post-transplant care	Patients in transplant units are followed-up routinely by multidisciplinary transplant teams. Some patients need to travel long distances for routine check-ups. There is no provision for shared-care protocols nor any infrastructure to support telemedicine
Operational Elements: Post-Transplant Follow-Up	National follow-up guidelines	National follow-up guidelines are lacking
	National outcomes monitoring	While short-term (1 year) graft and patient survival rates are monitored by the NTO, mid-term and long-term outcomes are not monitored
Training and Research: Research and Development	Research outputs	Minimal research is performed. Participation in international research efforts remains poor
	Research facilities and funding	There are no official research funding bodies. Access to research facilities (e.g., experimental laboratories, experimental surgery facilities) remains limited
Training and Research: Teaching, Training, and Professional Development	Continuous professional development for nurses and intensivists	Organ donation is not included as a core training module for intensivists or their support staff
	Continuous professional development for organ donation coordinators	Although the NTO has developed a sophisticated curriculum for training coordinators in cooperation with the Transplant Procurement Management-Donation and Transplantation Institute (TPM-DTI), this program is no longer operational
	Continuous professional development for physicians and transplant surgeons	There are no dedicated training programs for transplant surgeons or physicians
Professional Organizations and Scientific Societies	Engagement	Professional organizations and scientific societies are not involved in the development of national transplantation policy

### Enabling Elements

#### Government: Political Support, Funding, and Long-Term Commitment; Key Legislation

##### Inconsistent Political Support, Poorly Designed Legislation, and legislative Gaps Have Seriously Hindered Attempts at Reform

Political commitment and support for the Greek organ donation and transplantation program have always been inconsistent. The structural reforms of the early 2000s, including the foundation of the NTO and the launch of the ODC program, resulted in significant improvements in the donation and transplantation services available in Greece (38). Unfortunately, this initial improvement was not sustained over the long term due to a lack of financial stability, strategic planning, and continuity.

The 2008 national financial crisis disproportionally affected organ donation and transplantation activity in Greece (39). The austerity measures imposed at that time resulted in significant cuts to healthcare spending. Of note, during the years 2012–2017, the fraction of the GDP spent on healthcare in Greece decreased by 9.4% (5). This resulted in a severe restriction of the NTO’s budget and resulted in a dramatic downturn (i.e., a 60% decrease) in the rate of organ donation and transplantation (39).

In addition to adequate and consistent funding, political support designed to provide a consistent strategy for improvement will also be needed. In contrast to the roles and responsibilities of NTOs in many European countries (e.g., Italy, Portugal, Spain, and the United Kingdom [UK]), the Greek NTO can play only an advisory role with respect to policy development (6–10). Transplantation policy in Greece is developed by the Ministry of Health with input from the government; the NTO is not an independent authority and it cannot enact reforms (36). Thus, the national transplantation policy in Greece lacks a key pillar of stability and is thus vulnerable to political change.

Greece has developed extensive legislation focused on the definition and diagnosis of brain death. However, there is no separate legislation regarding the definition of brain death as distinct from its relationship with organ donation. This may ultimately create the false impression that a diagnosis of brain death is generated primarily to facilitate organ donation and transplantation. There is also ambiguity as to when to initiate this diagnostic process; the role of ancillary testing to confirm brain death is also poorly defined (35–37).

Greek law allows for organ donation after brain death (DBD) from adult and pediatric patients and for living donation (LD) from adult donors only (35–37). Pediatric LD is prohibited in Greece. Furthermore, there is no legislative provision for controlled or uncontrolled donation after circulatory death (cDCD or uDCD) from either adult or pediatric donors (35-37). As a result, there is no legislation in place that directs the practice of withdrawal of life-saving treatment (WLST) or a legal definition of “no-touch” time (i.e., the minimum time that must elapse between the confirmation of death and the commencement of measures needed to preserve organ viability). There is also no provision for directed or non-directed altruistic donation (DAD or NDAD) (35–37).

There have also been several poorly-planned changes in consent policy that have been introduced over the past decade. In 2011, the Greek government introduced an “opt-out” consent policy and provided 2 years for public information campaigns to take effect (40–42). However, there was little publicity regarding what these changes actually meant in practice for members of the general population (36–38). This resulted in a significant backlash against the new legislation, with many Greek citizens actively registering their objections to donation (i.e., opting out) (40–42). As a result, the “opt-in” consent policy was re-introduced in 2018 (33, 37). It is critical to recognize that many advanced European transplantation systems (e.g., those in Spain and the UK) include a “soft opt-out” policy in which donation can be pursued in the absence of specific documentation although family consent is required (6, 7).

#### Building and Maintaining Public Support and Trust in the System

##### Public Support for Organ Donation and Transplantation has Been Hampered by Mistrust, Limited Knowledge, and a Lack of Understanding

Public trust in the Greek National Organ Donation and Transplantation Program is limited compared to that enjoyed by its European neighbors. While 55% and 53% of Europeans agree in principle to personal or family-member organ donation, only 43% and 41% of Greeks agree with these principles, respectively. One of every two Greek citizens identifies distrust in the system as the primary reason for non-consent (43).

Awareness and understanding of organ donation among members of the general public also remains limited. In a survey conducted in 2019, Symvoulakis et al. (44–47) reported that only one of two respondents in a Greek rural population were aware of the possibility of deceased donation. Similarly, 7 of 10 Greek citizens report that they have never discussed organ donation (39); only 3.8% reported having some knowledge about the national processes and the existing legislative framework designed to promote organ donation (40–43). Of equivalent concern, most healthcare professionals in Greece reported that they did not consider themselves well-informed about organ donation (44–47).

In the past, organ donation has been promoted by mass media (26). Nevertheless, Greece does not have an ongoing, targeted communication strategy designed to promote organ donation and transplantation.

### Structural Elements

#### The National Transplant Organization (NTO)

##### The NTO is Severely Understaffed and Under-resourced and Cannot Drive Change

The Greek NTO lacks the leadership capacity needed to drive change. As outlined above, the Greek NTO has only an advisory role and lacks the authority to enact and follow through with substantial reforms. Recommendations suggested by the NTO can only be implemented upon approval by the national parliament or other political committees. In several of the European countries with successful transplantation systems (i.e., Italy, Portugal, Spain, and the UK), the NTOs are independent bodies with the capacity to enact change without the need for legislative recourse (6–10).

The organizational structure of the Greek NTO includes a president, a managing director, and three departments that are responsible for transplant coordination, management and finances, tissue storage, and histocompatibility testing (48). This structure does not correspond to the objectives and the responsibilities of the organization as defined by national legislation (49).

Furthermore, the Greek NTO is severely short-staffed and under-resourced. National law sets a minimum staffing requirement of eight temporary and 15 permanent employees, all of whom must have appropriate qualifications (36). At the time that this analysis was performed, the Greek NTO staff included only three permanent employees. The 15 locum positions are occasionally filled by professionals from different backgrounds; these positions experience high turnover. The NTO’s budget remains very restricted and currently does not provide adequate support for its functions (39).

On paper, the defined national and international responsibilities cover all the essential functions of an NTO that are identified by the conceptual framework (49). However, there was consensus among the interviewees that these responsibilities are not met in practice. However, we do note that Greece actively participates in several international transplant collaboration schemes (for example, collaboration with the Italian National Transplant Centre [CNT]) (50).

#### Infrastructure

##### Infrastructure and Human Resources Are not Adequate to Support the Program

There are five accredited transplant centers in Greece. A sixth facility, the Onassis National Transplant Center (ONTRC), is currently under construction with an expected completion date of 2024. However, there is little to no quantitative and qualitative data that address the human resources and infrastructure capacity of either Greek or other European transplant centers. To gain a better understanding of the resources available in these different settings, the research team surveyed all existing transplant centers in Greece as well as major transplant units in Croatia, Italy, Portugal, Spain, and the UK (26). Our results revealed that medical staffing levels in Greek transplant centers were below the average of these five European countries across all specialties (including surgeons, physicians, anesthetists, and others). There is also an urgent need to expand operating theater capacity for both donation and transplantation procedures and to improve access to imaging and other diagnostic services (e.g., endoscopy) across all existing units. Access to pathology services and histocompatibility testing must also be improved (26).

#### Reimbursement of Staff and Facilities

##### The Current System Does not Reimburse Organ Donation Activities or Adequately Compensate ODCs, Thus Creating Financial Barriers to Organ Donation

Creating financial incentives and ensuring that all parties involved (including staff and facilities) are adequately reimbursed and compensated for their work is a key feature of a successful transplantation system (6-10, 29). The diagnosis-related group reimbursement system (KEN-DRG) currently captures all transplantation activities in Greece and provides reimbursement to transplant units. However, the KEN-DRG does not reimburse donation activities (51). This discrepancy creates significant financial disincentives for donation units. Moreover, there is no legislative provision for the financial reimbursement of ODCs (52).

#### Registries, Databases, and Information Technology (IT)

##### There is Currently no Integrated IT System that Can be Accessed by all Personnel who are Tasked with Coordinating Organ Donation and Transplantation

Although the Greek NTO manages an organ donor registry, a non-donor registry, a DBD donor registry, and the national waiting lists, the lack of a centralized IT system is a key weakness as there is no mechanism in place that facilitates the timely and efficient exchange of information between the components of the donation and transplantation program.

#### Quality Standards and Continual Quality Improvement

##### There is an Urgent Need for Improved Quality Standards and a Clear Quality Improvement Strategy

National legislation has defined a series of quality standards that must be met prior to the authorization and licensing of transplant units (53). Similarly, the NTO is responsible for undertaking regular inspections (49, 53). However, we found no evidence of any regularly-scheduled inspections of transplant facilities, equipment, or personnel. No procedures designed to facilitate the improvement of organizations unable to meet expected standards have been established. At present, there are no quality standards in place that govern donation, pre-transplant, or post-transplant care.

A few quality indicators are monitored by the NTO and included in annual reports (13–24). While benchmarking performance data from each of the transplant centers are published every 3 years (13–24), these reports provided no evidence of quality improvement interventions.

### Operational Elements

#### Prevention

##### There are no Public Health Policies in Place Aimed at Reducing the Demand for Organ Transplantation

Greece has not adopted any public health programs designed to promote healthy diets (e.g., reduced consumption of salt, fat, and sugar), increased physical activity, limiting alcohol consumption, or smoking cessation. Greece has not promoted interventions designed to reduce the risk of hepatitis (e.g., prevention of intravenous drug use, avoiding high-risk sexual behaviors, undergoing immunization if possible) or to improve health literacy. High-risk populations are not screened for heart, lung, liver, and/or kidney disease. However, prompt access to tertiary prevention, most notably dialysis care, has been established (54). The quality of these tertiary prevention services is not monitored or evaluated on a regular basis.

#### Deceased Donation; Live Donation; Transplantation; Post-Transplant Follow-Up

##### There are Significant Gaps at all Stages of the Clinical Pathway, From Donation to Post-Transplant Follow-Up

Inadequate coordination of the donation process is one of the most important policy gaps identified in this report. The role of Organ Donation Coordinator (ODC) was first introduced in 1999 as a result of national legislation in Greece (36). The competencies, duties, and responsibilities of the role were clearly defined by this legislation. The ODC program was launched in 2005 with 55 coordinators who had received high-quality training (52, 55). Unfortunately, several factors intervened, including discontinuation of the training program, lack of protected time needed to perform duties, lack of financial compensation, and the perceived anti-social nature of working hours made the role very unpopular, and ODC recruitment became nearly impossible.

Another significant gap is the lack of national guidelines that can be used to inform the donation process. This includes guidelines for both deceased and living donor evaluation, organ retrieval, preservation, transport, and living donor follow-up. Several critical factors must be in place to support living donation, including the establishment of organ exchange schemes, the reimbursement of the financial losses of living donors, and the *a priori* prioritization of living donors on any transplant waiting list (in case of future need). These factors have all been enshrined in legislation to promote living donation (37). Despite this, Greece has not developed any national policies that raise public awareness and dispel misconceptions about living donations. Greece has also not focused on the creation of partnerships between referring specialists and the transplantation program that may be needed to promote living donation.

There are clear gaps in all the steps of the transplantation process. Beginning with pre-transplant care**,** there are no nationally agreed-upon criteria or processes for referral for assessment of suitability for transplant in Greece (1). Although deceased donor kidneys are allocated *via* a national waiting list using a universal point-based allocation system, Greece has no nationally agreed-upon, organ-specific criteria for prioritizing transplant listings.

The coordination of the transplant process itself is also deficient. Greece has only seven transplant recipient coordinators (TRCs), all of whom are based at the NTO headquarters in Athens, and are responsible for the coordination of all transplant procedures in the entire country. Because of the small number of TRCs, transplant coordination services are not available at all times.

With respect to operative and peri-operative care, feedback from the interviewees suggested that the lack of physical capacity (e.g., operating theatres) and human resources (e.g., surgeons and anesthetists) limits timely access to transplant procedures. There are no standardized national peri-operative care protocols in Greece.

There are also no national guidelines that direct follow-up care of transplanted patients (e.g., no directive regarding the frequency of follow-up or preferred immunosuppression strategies). Transplant recipients receive routine follow-ups from multidisciplinary teams in transplant units. There is no established system that permits local health centers to provide care. Thus, some patients need to travel long distances for routine follow-up appointments. This is a source of significant stress and unnecessary expense. Furthermore, mid-to long-term outcome data are not systematically collected or monitored (13–24).

### Training and Research

#### Teaching, Training, and Professional Development

##### There are Very Few Teaching, Training, or Professional Development Opportunities and no Official Training Programs for Healthcare Professionals

In the early 2000s, the NTO initiated a training program for ODCs, that was undertaken in part with cooperation with the Transplant Procurement Management-Donation and Transplantation Institute (TPM-DTI) in Barcelona (55). The training program featured a sophisticated curriculum that was in accordance with international best practices (29, 52). Unfortunately, there was little continuity and the program has since been abandoned; from 2006 to 2012, no training was available at all.

At the present time, Greece offers no official training programs (fellowships) for transplant surgeons and physicians. Individuals typically gain experience in transplantation during their subspecialty training (i.e., as part of their training in general surgery or general nephrology) or independent practice (i.e., at a European transplant center). Greece offers no official training in organ donation and transplantation for intensivists, anesthetists, or nurses.

#### Research and Development

##### Research and Development are not a Current Priority; the National Scientific Societies and Professional Bodies are not Involved in the Organ Donation and Transplantation Program

Transplantation research in Greece is not a priority; the resources allocated to medical research and development are below the European average (56). Since 1985, only 12 original research transplantation studies have been performed in academic institutions throughout the country (4). Greece participates only infrequently in international collaborative studies. There is insufficient high-level coordination of research activities in the field of organ donation and transplantation. There are no national research funding bodies or established pathways for researchers seeking funding. Moreover, the results of our survey demonstrated that transplant professionals in Greece have little to no access to the limited number of existing research facilities (26).

The national scientific societies (e.g., The Greek Transplantation Society, The Greek Society of Anesthesiology, and The Hellenic Society of Nephrology) and the professional bodies (e.g., the Greek Medical Association) are not engaged in the development of national transplantation policy and at present have no institutional or advisory roles.

### Recommendations

The full set of recommendations for reform of the Greek organ donation and transplantation program can be found in the Report for a New National Solid Organ Donation and Transplantation Plan in Greece (26). Due to the complex nature of this discipline, the recommendations cover many different aspects of the healthcare system and its governance in Greece. The overall intention of these recommendations is to align the organ donation and transplantation program with performance standards established by other European countries. Full implementation of some of the recommendations will necessitate wider health system reform. Thus, it is important to emphasize that while some components may seem to be more important than others, all are interconnected and interdependent. To achieve better rates of successful organ donation and transplantation, system-wide change will be critical. Neglect of one or more of the domains of the framework may result in ongoing underperformance. As a result, the successful implementation of these reforms will require strong commitment and collaboration between a wide range of stakeholders. It will also need strong top-down coordination accompanied by bottom-up engagement and implementation. This will require consistent support and commitment from the government.

The key recommendations which must be implemented if Greece hopes to improve its organ donation and transplantation program are outlined in the following sections. These sections reflect specific domains of the framework and the gaps identified in our analysis.

#### Government and Key Legislation

Long-term support from the central government will be needed to promote reform. Sustainable funding sources will need to be identified, and the NTO should be relaunched as an independent body with the authority to enact change.

Legislative reform will also be required to move toward a “soft opt-out” consent policy. This legislation must be complemented by public awareness campaigns and appropriate training of transplant professionals. Changes will be needed to separate the legislation focused on the neurological diagnosis of death and the principles of (DBD). Clinicians must receive regular training on the diagnosis of brain death, and the NTO must take the lead in establishing legislative provisions for DCD. A Pilot DCD program should be established in selected centers that already have the appropriate expertise and infrastructure.

#### Reducing the Need for Transplants, and Building and Maintaining Public Support and Trust

Policies aimed at reducing the need for transplantation must be developed. Primary, secondary, and tertiary preventative strategies must be devised and implemented.

Greece needs to build and maintain public support and trust in the transplant system by focusing on organ donation as a critical component of an altruistic society. Achieving this goal will require adherence to the strictest ethical standards, implementation of rolling public awareness and educational campaigns, and maintaining good relations with the media.

#### NTO, Databases and IT, Reimbursement, and Infrastructure

The re-launched, independent NTO must be appropriately staffed and provided with adequate funding. The recommendations provide clear and specific guidance for the governance, structure, and funding of the NTO and include recommendations for the establishment of a dedicated leadership team. A key responsibility of the relaunched Greek NTO will be to establish and maintain a national IT system with associated databases that can be used to improve the efficiency and effectiveness of the donation and transplantation program.

There is an urgent need to expand operating theater capacity (for both donation and transplantation procedures) and to improve access to imaging and other diagnostic services, for example, endoscopy. The KEN-DRG system must be revised to ensure that all activities related to organ donation and transplantation are adequately reimbursed, including the efforts of staff, participating units, and supporting services.

#### Patient-Centered Care

Transplant recipients (as well as their families and loved ones) and live donors must be the central focus of the national donation and transplantation program in Greece. Policies and procedures must be developed that promote collaborative engagement with patients, families, and caregivers, not only with respect to their own cases but also in all aspects of system planning and development.

#### Donation, Transplantation, and Follow-Up

The existing donation and transplantation workforce needs to be expanded. There is a particular need to increase the number of specially-trained ODCs who will then be appointed to every unit participating in organ donation. All processes along the clinical pathway, including patient follow-up, need to be subject to nationally agreed-upon protocols that are consistent with internationally-defined best practices. The NTO, alongside a panel of experts with organ-specific expertise, should be fully involved in generating this guidance.

Living donation (LD) is currently underutilized and should ultimately become a cornerstone of the donation and transplantation program in Greece. Pre-emptive renal transplantation from a living donor should become the treatment of choice for ESRD. Dialysis assessments must always involve discussions regarding the feasibility of transplantation and the possibility of identifying a living donor.

#### Quality Standards, Quality Improvement, and Scientific professional Organizations

The existing national system of quality assurance should be strengthened and expanded to include pre-transplant care. Existing quality indicators on donation and transplantation should be broadened and updated regularly. The NTO should develop additional capacity to support healthcare facilities seeking to reach compliance with regular audits based on key performance indicators conducted by the Body of Inspectors for Health and Welfare Services (SEYYP).

Professional organizations and scientific societies must be consulted at all stages of this programmatic expansion. The board of the NTO should include representatives from relevant organizations. These representatives should play pivotal roles in developing and ratifying guidelines, protocols, regulatory standards, and training programs for use throughout Greece.

#### Teaching, Training, and Professional Development

Greece must develop a national strategy that provides tailored and continuous training for healthcare professionals in all areas that are relevant for organ donation and transplantation. Dedicated training modules that follow existing guidelines created by the European Union of Medical Specialists should be available for use.

Improving the performance of the organ donation and transplantation system requires an innovative research and development program. All units and staff must be actively supported and encouraged to participate in research activities at local, national, and international levels.

#### Implementation Task Force

The implementation of the preceding recommendations will be a complex and time-consuming task. Thus, we recommend the establishment of an implementation task force that will be chaired by the permanent secretary of the Ministry of Health. We recommend the establishment of a 7–10-person task force that includes representatives from key interests. Annual reports focused on overall progress should be submitted for review.

## Discussion

This is the first comprehensive, systems-level study of the Greek organ donation and transplantation program published to date, and the first attempt to provide comprehensive recommendations for reform. Our findings were evaluated using a novel conceptual framework that takes a wide view of the field and attempts to capture all factors which might influence the success of a national donation and transplantation program. Thus, this study captures aspects of the Greek program that had not been considered in previous evaluations of Greece’s comparatively poor performance. Our study was also based on existing published and unpublished literature on solid organ donation and transplantation and was further informed by extensive rounds of interviews with numerous global, national, and local policy stakeholders in the field.

One limitation of this study was that the analysis was not designed to capture data focused on all aspects of a donation and transplant program (e.g., equity). We also recognize that the subjective views of the authors may have influenced the narrative review of the literature. Furthermore, we are aware that the views expressed by the interviewees may not be fully representative of all those working in the field of organ donation and transplantation in Greece.

Our study has highlighted numerous policy gaps with a significant impact on the national solid organ donation and transplantation program in Greece. A national reform program designed to address these policy gaps and implement the required policy changes should become a national priority. The recommendations provided represent a comprehensive set of potential policy priorities that can be used to strengthen the Greek national organ donation and transplantation system. Implementing these recommendations may accelerate Greece’s progress toward a national organ donation and transplantation program with performance metrics that are comparable to successful programs that have been developed in other European countries. This would deliver immeasurable improvements to the quality of life of thousands of individuals as well as their families.

As with all major policy reforms, the success of these efforts will rely on the manner of their implementation. One frequently observes a gap between policy plans and their implementation. Because of the extensive nature of the recommendations, coordination of the reforms will be a complex process. An implementation task force may play a crucial role in this endeavor and thus should have a formal mandate from the government to lead the policy design process and oversee the implementation of these reforms. The task force should also be provided with the responsibility of preparing the legislation needed to support the reform program as well as establishing a clear roadmap with due regard for the phasing of and interdependencies between the various programmatic elements. This roadmap should include clear targets and milestones that will ensure appropriate sequencing, for example, making certain that the capacity of the donation and transplantation workforce and infrastructure increases in concert with the coordination capacity of the system as well as increased rates of consent and availability of organs for transplant.

Finally, the task force should commit itself to rigor and transparency. For example, the task force might publish an annual report that highlights progress and identifies the gaps remaining to be addressed. Transparency will also be needed to support efforts to strengthen public support for organ donation and transplantation which will also be critical to the program’s success.

## Data Availability

The original contributions presented in the study are included in the article/Supplementary Material, further inquiries can be directed to the corresponding author.
