# Supplementary material for: The National Organ Donation and Transplantation Program in Greece: Gap Analysis and Recommendations for Change
Source: Transpl Int. 2023 May 25;36:11013. doi: 10.3389/ti.2023.11013 (PMC10249496; doi:10.3389/ti.2023.11013)
Supplement: Supplementary file 1 [file Table1.docx]

**Appendix 1**

| **Peer-Review Articles Identified from Literature Review** | | |
| --- | --- | --- |
| Author, Year | Title | Accessible through: |
| Sombolos K et al, 2014 | Multicenter Epidemiological Study to Assess the Population of CKD Patients in Greece: Results from the PRESTAR Study. | [https://doi.org/10.1371/journal.pone.0112767"10.1371/journal.pone.0112767](https://doi.org/10.1371/journal.pone.0112767%2210.1371/journal.pone.0112767) |
| Papoutsakis S., 2012 | The Era of Transplantations | <http://www.iedep.gr/images/stories/teuxi/issue31_1/The_Era_Transplantations.pdf> |
| Mavroforou A et al, 2004 | Organ and Tissue Transplantation in Greece: the Law and an Insight into the Social Context | <https://heinonline.org/HOL/LandingPage?handle=hein.journals/mlv23&div=15&id=&page=> |
| Karatzas T et al, 2007 | Substantial Increase in Cadaveric Organ Transplantation in Greece for the Period 2001–2005. | <https://www.sciencedirect.com/science/article/abs/pii/S0041134507003880?via%3Dihub> |
| Giorgakis E et al, 2018 | Transplantation crisis at the time of economic recession in Greece | <https://www.sciencedirect.com/science/article/abs/pii/S0033350618301288?via%3Dihub> |
| Moris D et al, 2016 | Organ donation during the financial crisis in Greece. | <https://www.thelancet.com/journals/lancet/article/PIIS0140-6736(16)30130-1/fulltext> |
| Sotiropoulos GC et al, 2016 | Correspondence to: Organ donation during the financial crisis in Greece. | <https://www.thelancet.com/journals/lancet/article/PIIS0140-6736(16)31488-X/fulltext> |
| Athanasios P, 2014 | Organ Transplants and “Presumed Consent”: laws 2737/1999 and 3984/2011, the National Experience and Future Trends | <https://www.vima-asklipiou.gr/en/articles/485/> |
| Bottis M, 2012 | The New Greek Statute on Organ Donation--Yet Another Effort to Advance Transplants | <https://brill.com/view/journals/ejhl/19/4/article-p391_6.xml> |
| Symvoulakis EK et al, 2009 | Attitudes to Kidney Donation Among Primary Care Patients in Rural Crete, Greece | <https://bmcpublichealth.biomedcentral.com/articles/10.1186/1471-2458-9-54> |
| Symvoulakis EK et al ,2012 | Kidney Organ Donation Knowledge and Attitudes Among Health Care Professionals: Findings from a Greek General Hospital | <https://www.sciencedirect.com/science/article/pii/S0897189712000262?via%3Dihub> |
| Symvoulakis EK et al,2013 | Organ Donation Awareness: Rethinking Media Campaigns | <https://www.ncbi.nlm.nih.gov/pmc/articles/PMC6358651/> |
| Symvoulakis EK et al,2013 | Shifting towards an opt-out system in Greece: a general practice-based pilot study | <https://www.ncbi.nlm.nih.gov/pmc/articles/PMC3775113/> |
| Peritore D et al, 2012 | Italy-Greece Cooperation for Transplantation of Medically Urgent Greek Patients: Is It an Effective, Efficient model? | <https://www.sciencedirect.com/science/article/abs/pii/S0041134512005921?via%3Dihub> |
| Karatzas T et al, 2007 | Improving the Organ Transplantation Program in Greece: Institution of Local Transplant Coordinators’ Network | <https://www.sciencedirect.com/science/article/abs/pii/S0041134507004940?via%3Dihub> |
