# Supplementary material for: The National Organ Donation and Transplantation Program in Greece: Gap Analysis and Recommendations for Change
Source: Transpl Int. 2023 May 25;36:11013. doi: 10.3389/ti.2023.11013 (PMC10249496; doi:10.3389/ti.2023.11013)
Supplement: Supplementary file 2 [file Table2.docx]

**Appendix 2**

**Stakeholders consulted**

**National Transplant Organization**

Professor George Papatheodoridis, President

Ms Yiouli Menoudakou, Head of Transplant Co-ordination

Mr. Fotis Antoniou, Transplant Co-ordinator, Thessaloniki

Ms. Dimitra Zacharoudi, Transplant Co-ordinator, Thessaloniki

**Hellenic Medical Association**

Dr. Athanasios Exadaktylos, President

**Histocompatibility Laboratories**

Professor Aliki Iniotaki, former Director, Immunology Laboratory and National Tissue Typing

Center, General Hospital of Athens G. Gennimatas

**National Bioethics Committee**

Professor Eleni Rethymiotaki

**Transplant Centers**

Dr. Stamatis Adamopoulos, Cardiologist, Onassis Cardiac Surgery Center

Dr. Nikolaos Antoniadis, Transplant Surgeon, Ippokrateio General Hospital of Thessaloniki

Dr. Ioannis Bokos, Transplant Surgeon, Laiko

Professor Ioannis Boletis, Nephrologist, Laiko

Professor Dimitris Goumenos, Nephrologist, University Hospital of Patras

Professor Dionysios Karavias, Transplant Surgeon, University Hospital of Patras

Dr. Michail Mitsis, Transplant Surgeon, University Hospital of Ioannina

Professor George Tsoulfas, Transplant Surgeon, Ippokrateio General Hospital of Thessaloniki

Dr Vasileios Vougas, Transplant Surgeon, Evagelismos

**Intensive Care Units**

Dr. Nikolaos Kapravelos, Papanikolaou General Hospital of Thessaloniki

Dr. Iraklis Tsagkaris, Laiko

**Scientific Societies**

Professor Ioannis Boletis, Hellenic Society of Nephrology

Professor Anastasia Kotanidou, Hellenic Transplantation Society

Dr. Anna Malisiova, Hellenic Society of Anaesthesiology

**World Health Organization**

Mr. Stratos Chatziksiros, Member

**Patient Associations**

Mr. Nikos Karafolas, Heart and Lung Transplant Patients’ Association

Mr. Grigorios Leontopoulos, Renal Transplant Patients’ Association

Ms. Christina Theodoridou, Liver Transplant Patients’ Association
